# SH2 Ligand-Like Effects of Second Cytosolic Domain of Na/K-ATPase α1 Subunit on Src Kinase

**DOI:** 10.1371/journal.pone.0142119

**Published:** 2015-11-09

**Authors:** Moumita Banerjee, Qiming Duan, Zijian Xie

**Affiliations:** 1 Marshall Institute for Interdisciplinary Research (MIIR), Marshall University, Huntington, West Virginia, United States of America; 2 Case Cardiovascular Research Institute, Department of Medicine, Case Western Reserve University School of Medicine and Harrington Heart and Vascular Institute, Cleveland, Ohio, United States of America; Seoul National University, REPUBLIC OF KOREA

## Abstract

Our previous studies have suggested that the α1 Na/K-ATPase interacts with Src to form a receptor complex. *In vitro* binding assays indicate an interaction between second cytosolic domain (CD2) of Na/K-ATPase α1 subunit and Src SH2 domain. Since SH2 domain targets Src to specific signaling complexes, we expressed CD2 as a cytosolic protein and studied whether it could act as a Src SH2 ligand in LLC-PK1 cells. Co-immunoprecipitation analyses indicated a direct binding of CD2 to Src, consistent with the *in vitro* binding data. Functionally, CD2 expression increased basal Src activity, suggesting a Src SH2 ligand-like property of CD2. Consistently, we found that CD2 expression attenuated several signaling pathways where Src plays an important role. For instance, although it increased surface expression of Na/K-ATPase, it decreased ouabain-induced activation of Src and ERK by blocking the formation of Na/K-ATPase/Src complex. Moreover, it also attenuated cell attachment-induced activation of Src/FAK. Consequently, CD2 delayed cell spreading, and inhibited cell proliferation. Furthermore, these effects appear to be Src-specific because CD2 expression had no effect on EGF-induced activation of EGF receptor and ERK. Hence, the new findings indicate the importance of Na/K-ATPase/Src interaction in ouabain-induced signal transduction, and support the proposition that the CD2 peptide may be utilized as a Src SH2 ligand capable of blocking Src-dependent signaling pathways via a different mechanism from a general Src kinase inhibitor.

## Introduction

Signal transduction in general relies on regulated, coordinated and dynamic protein-protein interaction. The signaling pathways involving Src kinase are among the well-studied, because Src deregulation has been linked to the development and progression of many diseases including cancer and chronic kidney diseases. Src is a non-receptor tyrosine kinase, known to regulate cell proliferation, adhesion and migration [1–3]. It consists of two well defined protein-protein interaction sequences named Src homology domain 2 and 3 (SH2 and SH3). The SH2 domain is a module containing about 100 amino acids, and binds to a specific phosphotyrosine sequence in the target protein [4, 5]. While myrisoylation and palmitoylation plays an essential role for targeting of Src to the plasma membrane, the SH2- and SH3-mediated protein interaction allows Src binding to specific signaling complexes in the plasma membrane [6–9]. Because the SH2-mediated protein interaction is important for the activation and targeting of Src, significant efforts have been made to develop Src SH2 ligand as an alternative way of regulating Src-mediated signal transduction in addition to kinase inhibitors [10, 11].

Na/K-ATPase is a ubiquitously expressed membrane protein that transports Na^+^ and K^+^ ions in and out of the cells at the expense of ATP hydrolysis. It contains two major subunits–a α subunit which is responsible for transporting ions and a β subunit which is responsible for targeting of the protein to the plasma membrane [12, 13]. We have previously reported the discovery of a Na/K-ATPase/Src receptor complex that could allow cardiotonic steroids such as ouabain to activate protein kinase cascades such as the Ras/Raf/ERK [14, 15]. We have further demonstrated a direct interaction between the N domain of α1 subunit of Na/K-ATPase and Src kinase domain [16, 17]. In accordance, we have mapped the Src kinase domain binding motif in the α1 N domain and developed a 20 amino acid residue peptide called pNaKtide that specifically inhibits Na/K-ATPase-associated Src signaling [18]. Our *in vitro* GST-pull down assay also revealed a potential interaction between the second cytosolic domain (CD2) of α1 subunit of Na/K-ATPase and Src SH2 domain [14, 16]. As the first step to study this interaction in live cells, we made an YFP-CD2 expression vector based on our *in vitro* GST-pull down assay data [14], transfected it into LLC-PK1 cells, and generated several stable cell lines. This allowed us to look at the possible functional interaction between CD2 and Src in live cells without the interference from the interaction between Src kinase domain and α1 N domain [18, 19]. The new findings are consistent with the notion that CD2 could interact with Src directly and affects Src-mediated signal transduction like a SH2 ligand.

## Materials and Methods

### Materials

The following antibodies were obtained from Santa Cruz Biotechnology (Santa Cruz, CA)- monoclonal anti-Src antibody (B12), polyclonal anti-ERK1/2 (Extracellular Regulatory Kinase 1 /2) antibody, monoclonal anti-phospho ERK1/2 antibody, monoclonal anti-FAK (Focal adhesion kinase) antibody, goat anti-mouse IgG HRP and goat anti-rabbit IgG HRP secondary antibodies. Monoclonal anti-His, anti-Src (pY418) and (pY529) polyclonal antibodies were from Invitrogen (Carlsbad, CA). Anti-GFP (Green Fluorescent Protein) rabbit polyclonal antibody was from Abcam (Cambridge, MA). Anti-phospho EGFR (pY1173), anti-EGFR antibodies and anti-phospho FAK (pY576/577) rabbit polyclonal antibody were from Cell Signaling Technologies (Danvers, MA). The monoclonal anti-α1 Na/K-ATPase subunit antibody (α6f) was obtained from Developmental Studies Hybridoma Bank at The University of Iowa (Iowa City, IA). Texas Red-X phalloidin was from Invitrogen. Monoclonal anti-Src GD11 antibody, purified recombinant human Src and polyclonal anti-Na/K-ATPase α1 antibody were obtained from Upstate Biotechnology (Lake Placid, NY). The CMV promoter driven pEYFP-C1 vector was purchased from Clontech (Palo Alto, CA). Transfection kits were Lipofectamine 2000 and Lipofectamine PLUS LTX from Invitrogen. Glutathione beads were purchased from Amersham Biosciences (Uppsala, Sweden) and Probond purification system was from Invitrogen. All other reagents were purchased from Sigma-Aldrich (St. Louis, MO).

### Plasmid Constructs

Plasmid construct bearing GST (Glutathione S Transferase) fusion protein CD2 (amino acid residues 151–286, Swiss Prot ID-P05024) was prepared as previously described [20]. EYFP (Enhanced Yellow Fluorescent Protein)-tagged CD2 construct was generated by PCR amplifying the CD2 segment and inserting it at the C terminus of EYFP in pEYFP-C1 vector. All constructs were verified by DNA sequencing.

### Cell culture and generation of different cell lines

Pig kidney epithelial LLC-PK1 cells [21] and mouse fibroblast SYF and SYF+Src cells [22] were purchased from ATCC (Mannassas, VA) and cultured in DMEM (Dulbecco’s Modified Eagles Medium) containing 10% (v/v) FBS (fetal bovine serum) with 100U/ml penicillin and 100μg/ml streptomycin in a 5% CO_2_-humidified chamber. LLC-PK1 cells were allowed to reach 95–100% confluence and then serum starved overnight for experiments. To generate stable cell lines, LLC-PK1 cells were transfected (Lipofectamine 2000) with pEYFP-C1 vector containing CD2 construct or pEYFP-C1 empty vector. After verifying YFP expression visually, the cells were selected with 1mg/ml G418 for one week. G418 resistant colonies were selected and expanded. Cells were then cultured without G418 for at least three generations before being used for experiments. SYF and SYF+Src cells were transiently transfected with pEYFP-C1 or pEYFP-C1-CD2 construct using Lipofectamine PLUS LTX reagent. Transfection efficiency was above 50% for both SYF and SYF+Src cells.

### Immunoprecipitation and immunoblot analysis

Cells were washed with ice-cold PBS (Phosphate-buffered saline), and then solubilized in modified radio immunoprecipitation assay buffer containing 0.25% sodium deoxycholate, 1% Nonidet P-40, 1mM EDTA, 1mM phenylmethylsulfonyl fluoride, 1mM sodium orthovanadate, 1mM NaF, 10μg/ml aprotinin, 10μg/ml leupeptin, 150mM NaCl, and 50mM Tris-HCl (pH 7.4). Cell lysates were centrifuged at 14,000rpm for 15 minutes; supernatants were collected and subjected to immunoprecipitation or Western blot analysis as described [14].

### ^3^H-Ouabain binding assay and Ouabain-sensitive ^86^Rb^+^ uptake

^3^H-Ouabain binding assay was performed as described previously [20]. The transport function of Na/K-ATPase was assessed by measuring the ouabain-sensitive uptake of the K^+^ congener, ^86^Rb^+^, as described [20] with minor modifications. Briefly, cells were cultured in 12-well plates over 90% confluence and serum starved overnight before experiment. The cells were washed and incubated in culture medium with or without ouabain (1mM) over 10 minutes at 37°C. Monensin (20μM) was added to clamp extracellular Na^+^ to ensure maximal capacity of active uptake. ^86^Rb^+^ (1μCi/well) as a tracer for K^+^, was then added for 10 minutes at 37°C and the reaction was stopped by washing three times with ice-cold 0.1M MgCl_2_. Then cells were incubated in 10% trichloroacetic acid (TCA) and TCA soluble ^86^Rb^+^ was counted in a Beckman scintillation counter. TCA-precipitated proteins were dissolved in 0.1N NaOH and 0.2% SDS solution and the concentration was determined using the BioRad Protein Assay Kit (BioRad Laboratories, Hercules. CA). All counts were normalized to protein amount.

### Cell proliferation assay

Cell proliferation assay was performed as described previously [23]. Briefly, 20,000 cells/well were seeded in triplicates in 12 well plates in DMEM containing 10% FBS. At indicated time points, cells were trypsinized and counted.

### Cell spreading assay

Cell spreading assay was performed as described by Richardson et al [24]. Cells were harvested by trypsinization and 200,000 cells were plated in 60 mm dish containing 4 ml of DMEM with 10% FBS. Cells were then allowed to spread at 37°C for the indicated time points. Images of cell spreading were recorded by phase contrast microscope and for each experiment 5 random fields were photographed. At least 300 cells per experimental condition were counted. Spread cells are defined as those which had extended processes, lacked a rounded morphology and were not phase bright. In order to analyze the spreading of transfected SYF and SYF+ Src cells, 400,000 cells were seeded in 60 mm dishes and cell spreading was recorded using a fluorescent microscope at indicated time points. Spreading of untransfected SYF and SYF+Src cells were measured using a phase contrast microscope.

### Cell spreading-associated kinase activity assay

Dishes (100 mm) were coated overnight with 10ug/ml fibronectin (in PBS) at 4°C. Cells were grown up to 90% confluence and serum starved with DMEM + 0.5% FBS for 24 hours. On the day of the experiment, dishes were first washed with PBS solution and incubated with serum free media at 37°C for 1 hour. Cells were harvested using 0.05% trypsin + 0.53mM EDTA. The trypsin was then neutralized by adding 0.5mg/ml of Soybean Trypsin inhibitor in PBS. The cells were then washed, suspended in serum free medium and incubated in 15ml Falcon tubes for 1 hour at 37°C. An aliquot of cells (6,000,000) were plated in the fibronectin-coated dishes and allowed to attach/spread for 0, 30 or 60 minutes. At the indicated time points, dishes were removed from incubator, washed once with ice-cold PBS, and attached cells were lysed in ice-cold lysis buffer as previously described [25]. Cell lysates were collected and analyzed by Western blot.

### Confocal microscopy

Cells were seeded on coverslips and allowed to grow to 90% confluence. Cells were then fixed with 3.7% formaldehyde for 5 minutes followed by permeabilization with 10% Triton X-100 for 10 minutes. The cells were then washed once with PBS, and blocked in PBS containing 1% goat serum for 30 minutes. Coverslips were again washed with PBS and incubated with 1:100 solution of Texas Red-conjugated Phalloidin in PBS solution for 1 hour at room temperature. Coverslips were washed twice and mounted on slides with Prolong Gold Antifade Reagent (Invitrogen, CA). Images were taken using a Leica DMIRE2 confocal microscope (Germany). Quantitative analyses of actin stress fibers were performed using ImageJ software from NIH (Bethesda, MD).

### GST tagged protein purification and binding assay

GST, GST-tagged CD2 and His-tagged Src SH2SH3 domain proteins were expressed and purified as described [14]. *In vitro* assay was performed by incubating Glutathione bead-conjugated GST or GST-CD2 with different amount of His-tagged SH2SH3 domain in PBS, for 30 minutes at room temperature. The beads were then washed thoroughly with PBS in the presence of 0.5% Triton X-100 four times. The bound SH2SH3 was then resolved on SDS-PAGE and detected by Western blot using anti-His monoclonal antibody.

### Data analysis

Data are given as mean ± SEM. Statistical analysis were performed using Students T test and significance was accepted at p<0.05.

## Results

**Fig 1:**
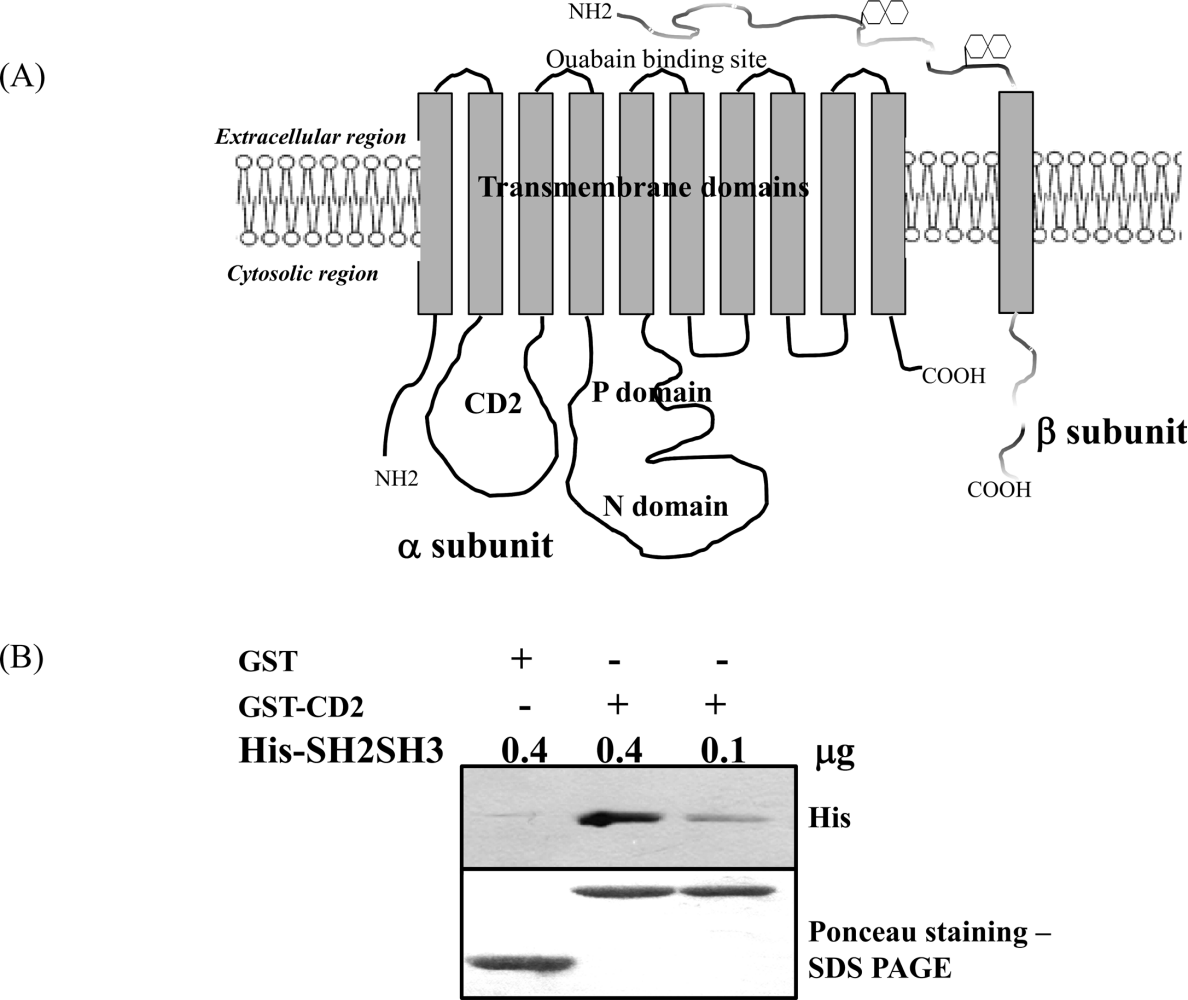
(A) A schematic diagram of Na/K-ATPase with all the important domains is shown. CD2-second cytosolic domain, P domain-phosphorylation domain and N domain- nucleotide binding domain. (B) GST pull down analysis showing a dose-dependent interaction between CD2 and His-SH2SH3. The upper panel shows the Western blot of anti-His, and the lower panel shows Ponceau staining of GST and GST-CD2. A representative Western blot from three independent experiments is shown.

The overall topology of α1 subunit of Na/K-ATPase is comprised of ten transmembrane domains (TM1-10), and the amino and carboxy termini are both cytosolic (Fig 1A). The cytosolic segment connecting TM2 and TM3 is shown and labeled as CD2. The data presented in Fig 1B confirmed our prior in vitro binding result [14], showing a dose-dependent high affinity binding of His-tagged Src SH2SH3 to GST-CD2. Because Src SH3 does not interact with Na/K-ATPase [14], these findings indicate a direct interaction between Src SH2 and CD2.

### Generation and characterization of CD2 expressing cell lines

**Fig 2:**
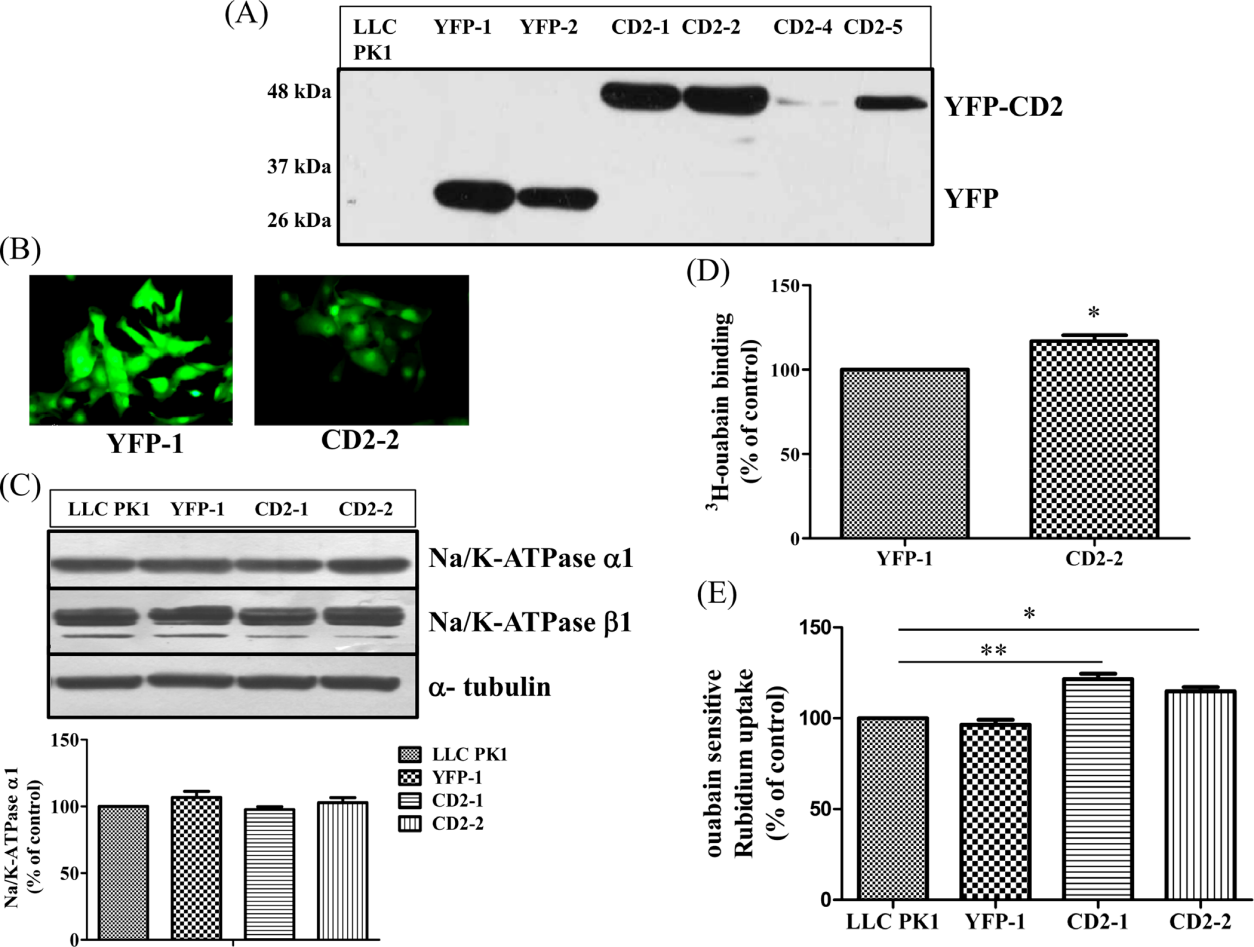
Generation and characterization of CD2 expressing cells (A) Stable cell lines expressing either YFP (YFP-1 and YFP-2) or YFP tagged CD2 (CD2-1, CD2-2, CD2-4 and CD2-5), were created from pig kidney epithelial cell line LLC-PK1. Equal amount of cell lysates from all the seven cell lines were analyzed by Western blot for YFP expression. (B) Fluorescent images of two representative cell lines are shown (images were taken at the same scale). (C) Na/K-ATPase α1 and β1 subunit expression. Total cell lysates from LLC PK1, YFP-1, CD2-1 and CD2-2 cell lines were analyzed for expression of α1 and β1 subunit of Na/K-ATPase. Representative western blots are shown and quantitative data are from at least three independent experiments. As loading control, α-tubulin was probed on the same blot. (D) Cells were grown up to 100% confluence and ouabain binding was measured according to protocol described in “Materials and Methods” section. The values are mean± SEM from at least three independent experiments.* p<0.05 compared with control. (E) Ouabain-dependent ^86^Rb^+^ uptake was measured as described in the “Materials and Methods”. Values are mean± SEM from at least three independent experiments. * p<0.05 compared with LLC-PK1, **p<0.01 compared with LLC-PK1.

Our in vitro binding data indicate that CD2 could bind Src SH2 with high affinity (Fig 1B)[14, 16]. In order to test whether CD2, when expressed as a cytosolic protein, could function as a Src SH2 ligand, we made several stable cell lines that express YFP-CD2 fusion protein. Cells were transfected with either pEYFP-C1 or pEYFP-C1-pig α1 CD2 vector, and selected by G418. Numerous G418 resistant clones were generated. After both Western blot analyses and fluorescence microscopic imaging, we picked and expanded four stable cell lines (CD2-1, CD2-2, CD2-4 and CD2-5) that expressed different amount of YFP-CD2 (Fig 2A). We also generated two stable empty vector-transfected cell lines (YFP-1 and YFP-2) for use as controls. As depicted in Fig 2A, clone CD2-2 expressed the highest amount of YFP-CD2, followed by clones CD2-1, CD2-5 and CD2-4. The same experiments showed that YFP expression was higher in YFP-1 cells than that in YFP-2 cells. Thus, YFP-1 and CD2-2 cell lines were used for most of the following experiments, and other cell lines were used to verify the main findings or as a control.

To assess the cellular distribution of the expressed YFP-CD2, fluorescence microscopic images were taken, showing that YFP-CD2 and YFP had similar pattern of cellular distribution as soluble proteins (Fig 2B).

To test whether the expression of YFP-CD2 altered the pumping function of Na/K-ATPase, we first determined the expression of Na/K-ATPase α1 subunit. As shown in Fig 2C, there was no detectable difference between the control and CD2 expressing cells. However, an 16% increase in the surface expression of α1 Na/K-ATPase as measured by ^3^H-Ouabain binding assay was noted in CD2-2 cells as compared with that in YFP-1 cells (Fig 2D). This was consistent with about ~15% increase in ouabain-sensitive ^86^Rb^+^ uptake in CD2-2 cells, and further confirmed in CD2-1 cells (Fig 2E).

### CD2 as a potential SH2 ligand

**Fig 3:**
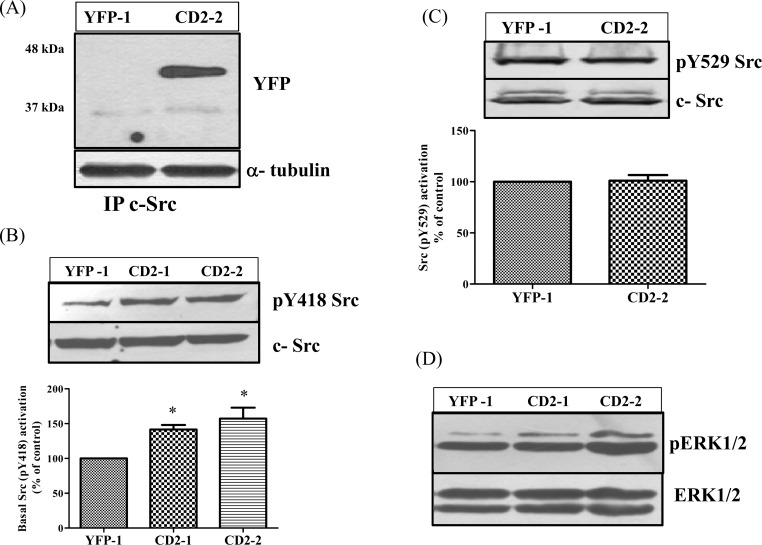
CD2 binds and activates Src kinase. (A) Five hundred μg of total cell lysate was immunoprecipitated with 3μg anti-Src antibody and immunoprecipitates were subjected to Western blot analysis of YFP and Src. A representative blot of at least three independent experiments is shown. (B) YFP-1, CD2-1 and CD2-2 cells were grown up to 90% confluence, serum-starved overnight and total cell lysates were analyzed for pY418 Src and total cellular Src (c-Src). A representative Western blot is shown and the data are mean± SEM of at least three independent experiments. * p<0.05. (C) Total cell lysates were analyzed for pY529 Src and cellular Src (c-Src) in the same way as described in B. Quantitative analysis of at least three independent experiments is shown. (D) Cell lysates from different cells were analyzed for phospho- ERK1/2 and total ERK1/2. A representative Western blot is shown.

To test whether CD2 interacts with Src in live cells, we first conducted a co-immunoprecipitation analysis. Cell lysates from both control YFP-1 and CD2-2 cells were immunoprecipitated by a monoclonal anti-Src antibody. Immunoprecipitates were then analyzed by Western blot using anti-Src and anti-GFP antibodies. As shown in Fig 3A, anti-Src antibody co-precipitated YFP-CD2, but not YFP, in the cell lysates, consistent with the previous *in vitro* GST-pull down assays (Fig 1) [14, 16].

To test whether CD2 could act as a Src SH2 ligand, interacting and regulating Src activity, we measured basal Src activity in CD2-2 and the control YFP-1 cell lines. Should CD2 interact with the Src SH2, we would expect an increase in basal Src activity because such an interaction could displace the intra-molecular SH2/pY529 interaction, resulting in Src activation as shown by other SH2 ligands [26, 27]. Indeed, as depicted in Fig 3B, the expression of YFP-CD2 increased basal Src activity in CD2-2 cells as measured by Western blot analyses of Src tyrosine phosphorylation at Y418 (pY418). This was further confirmed in CD2-1 cells. When the phosphorylation of Y529 was measured, there was no detectable difference in pY529 between the control and CD2-2 cells (Fig 3C), indicating that the increase in Src activity by the expression of YFP-CD2 is not because of a decrease in Y529 phosphorylation. Because ERKs are known downstream effectors of Src activation, we also measured ERK activity in the cell lysates. An increase in ERK activity was noted (Fig 3D).

### Inhibition of Na/K-ATPase/Src interaction

**Fig 4:**
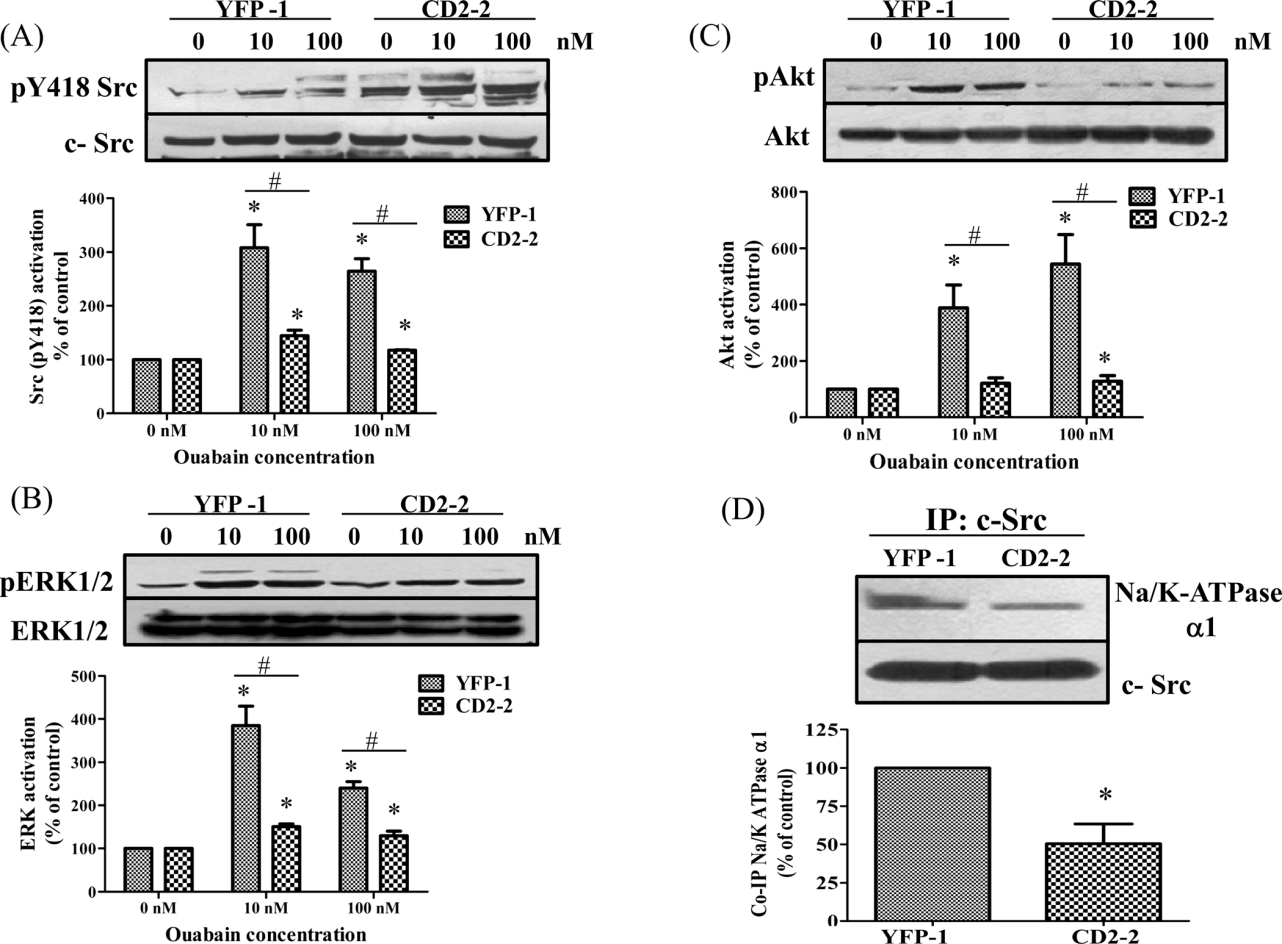
Effects of CD2 on ouabain-induced signal transduction Cell lines were treated with indicated concentrations of ouabain for 10 minutes. Cell lysates were collected and analyzed for A) pY418 Src and total cellular Src (c-Src), B) phospho-ERK1/2 and total ERK1/2 C) phospho- Akt and total Akt. Representative Western blots are shown and quantitative data are mean± SEM of at least three independent experiments. * p<0.05, compared with 0nM control, #, p<0.05, compared with different cell line. D) Five hundred μg of total cell lysate was immunoprecipitated with 10μg of anti-Src antibody and analyzed for co-precipitated Na/K ATPase α1. A representative western blot is shown. Values are mean ± SEM from at least three independent experiments. * p<0.05 compared with control.

We have proposed that the interaction between the α1 CD2 and Src SH2 is important for the formation of a functional Na/K-ATPase/Src receptor complex [14, 16]. It is known that ouabain activates Src and ERK in LLC-PK1 cells through the functional Na/K-ATPase/Src receptor complex [28]. Thus, to further address the functionality of YFP-CD2, we exposed the control YFP-1 and CD2-2 cells to different concentrations of ouabain and then measured the activity of cellular Src and ERK. As depicted in Fig 4A and 4B, expression of CD2 significantly attenuated ouabain-induced Src and ERK activation even through the surface expression of Na/K-ATPase was actually increased in the CD2-2 cells (Fig 2D and 2E).

It is known that ouabain also stimulates PI3K/Akt pathways in LLC-PK1 and other cells, and that the activation of Src is important for the full activation of Akt [20, 29]. To further verify the inhibitory effects of YFP-CD2 on ouabain-induced signal transduction, we exposed both control and CD2-2 cells to different concentrations of ouabain and measured Akt activation by Western blot analysis of cell lysates. As depicted in Fig 4C, ouabain-induced Akt activation was significantly attenuated by the expression of YFP-CD2. Thus, YFP-CD2 appears to work as a dominant negative mutant, capable of blocking ouabain-induced signal transduction including the activation of Src, ERKs and Akt.

To probe the molecular mechanism of the observed inhibition, we immunoprecipitated Src using a monoclonal anti-Src antibody from CD2-2 cell lysates and measured co-precipitated α1 subunit of Na/K-ATPase. Cell lysates from control YFP-1 cells were subjected to the same measurements and used as a control. As depicted in Fig 4D, expression of YFP-CD2 significantly reduced the co-precipitated α1 subunit of Na/K-ATPase from CD2-2 cell lysates in comparison to that in YFP-1 cell lysates, which is in accordance with the finding that YFP-CD2, not YFP, co-precipitated with Src as depicted in Fig 3A. Thus, while the expression of CD2 increased surface expression of Na/K-ATPase, it actually reduced the formation of receptor complex (Na/K-ATPase/Src), leading to the inhibition of ouabain-induced signal transduction in CD2-2 cells.

To verify that the inhibition of ouabain signaling in CD2-2 cells was not due to the selection of CD2-2 clone, we also exposed the CD2-1 cells to ouabain and measured ERK activation. Ouabain-induced ERK activation was also attenuated in CD2-1 cells (S1 Fig).

### Expression of YFP-CD2 inhibits Src-mediated integrin/FAK signaling

**Fig 5:**
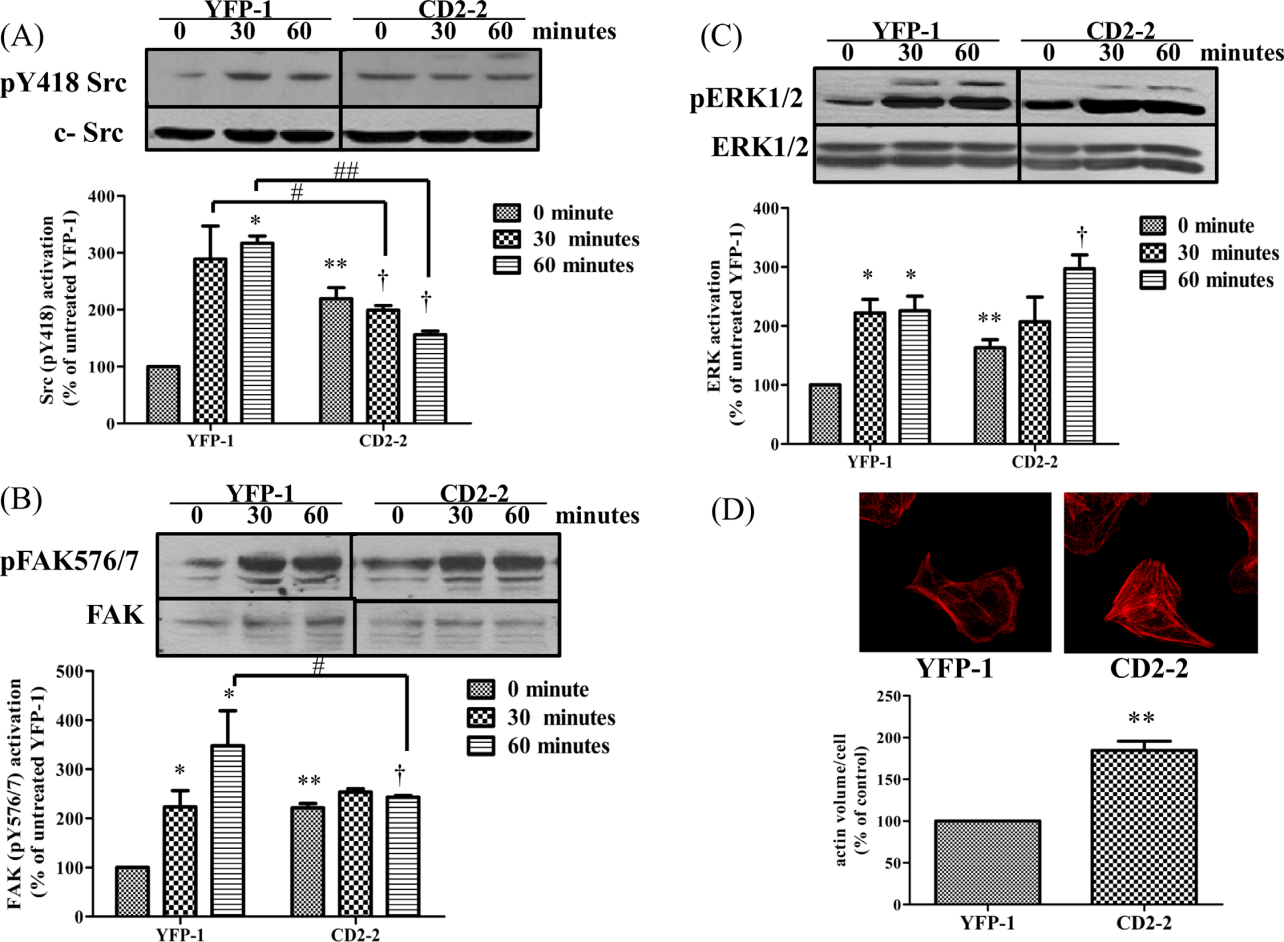
Effects of CD2 on FAK/Src signaling. Serum-starved YFP-1 and CD2-2 cells were harvested and plated for indicated time points as described under “Materials and Methods”. Total cell lysates were analyzed for (A) pY418 Src/total Src, (B) pFAK 576/577/total FAK, and (C) pERK/total ERK. Representative Western blots are shown and quantitative data are mean± SEM of at least three independent experiments. * p<0.05 compared with untreated YFP-1, ** p<0.01 compared with untreated YFP-1, # p<0.05 compared among different cell lines,## p<0.01 compared among different cell lines, † p<0.05 compared with untreated CD2 cells. (D) To stain for actin cytoskeleton, cells were grown up to confluence on coverslips and prepared for imaging as described in “Materials and Methods”. Images were taken at the same setting. A representative set of images from four separate experiments is shown. Quantitative analysis (with ImageJ) from 200 cells is shown. **p<0.01 compared with control.

To further test the inhibitory activity of CD2 as a general Src SH2 ligand on Src-mediated signal transduction, we examined whether the expression of CD2 alters FAK signaling where Src recruitment and binding via the SH2 domain is essential [30–33]. We plated both control YFP-1 and CD2-2 cells in fibronectin-coated dishes and then measured the attachment-induced time-dependent changes in Src/FAK activity. As shown in Fig 5A, a time-dependent activation of Src was observed in control cells after plating. This activation was significantly reduced in CD2-2 cells. Moreover, when FAK, a known Src effector in integrin signaling [31], was probed for tyrosine phosphorylation, an increase in Y576/577 phosphorylation was observed in control cells (Fig 5B). Again, this increase was attenuated in CD2-2 cells. In accordance with the defects in Src and FAK signaling, CD2-2 cells contained more stress fibers than YFP-expressing cells (Fig 5D). Interestingly, when ERK activation was assessed, we found that cell attachment-induced ERK activation was not reduced by the expression of YFP-CD2 (Fig 5C).

#### CD2 has no effect on EGF-induced receptor tyrosine phosphorylation and ERK activation

**Fig 6:**
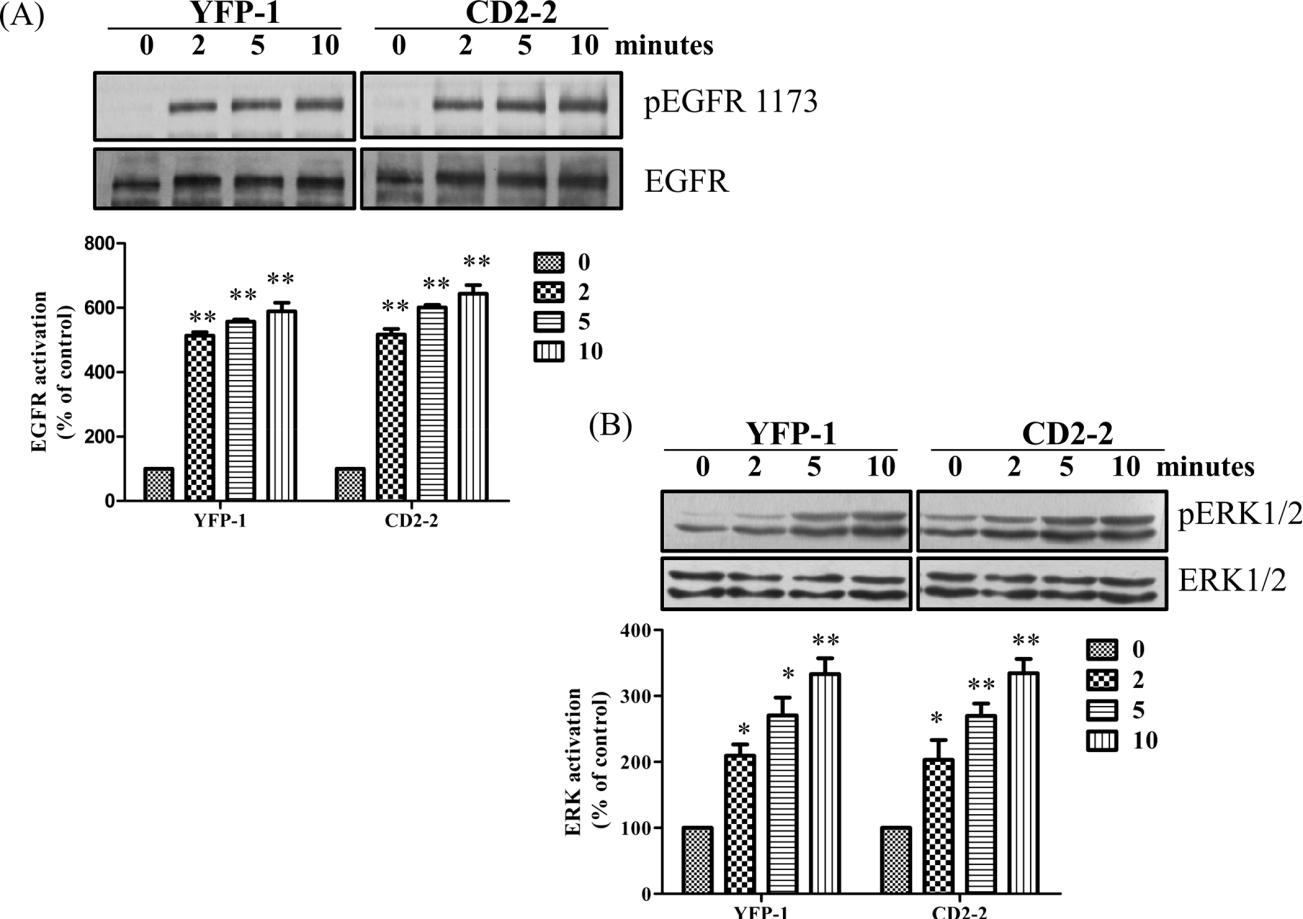
CD2 expression has no effect on EGF induced EGFR and ERK phosphorylation- YFP-1 and CD2-2 cells were treated with 10ng/ml of EGF for the indicated time points. Cell lysates were analyzed for A) phospho-EGFR 1173 / total EGFR and B) phospho-ERK1/2/total ERK 1/2. Representative Western blots are shown and quantitative data are from three independent experiments. Values are mean ± SEM. * p<0.05 compared with control, ** p<0.01 compared with control.

To further test the specificity of CD2-induced inhibition of cell signaling, we exposed both control and CD2-2 cells to EGF. As depicted in Fig 6A and 6B, EGF-induced receptor tyrosine phosphorylation and ERK activation were not affected by CD2 expression.

### CD2 inhibits cell spreading and growth

**Fig 7:**
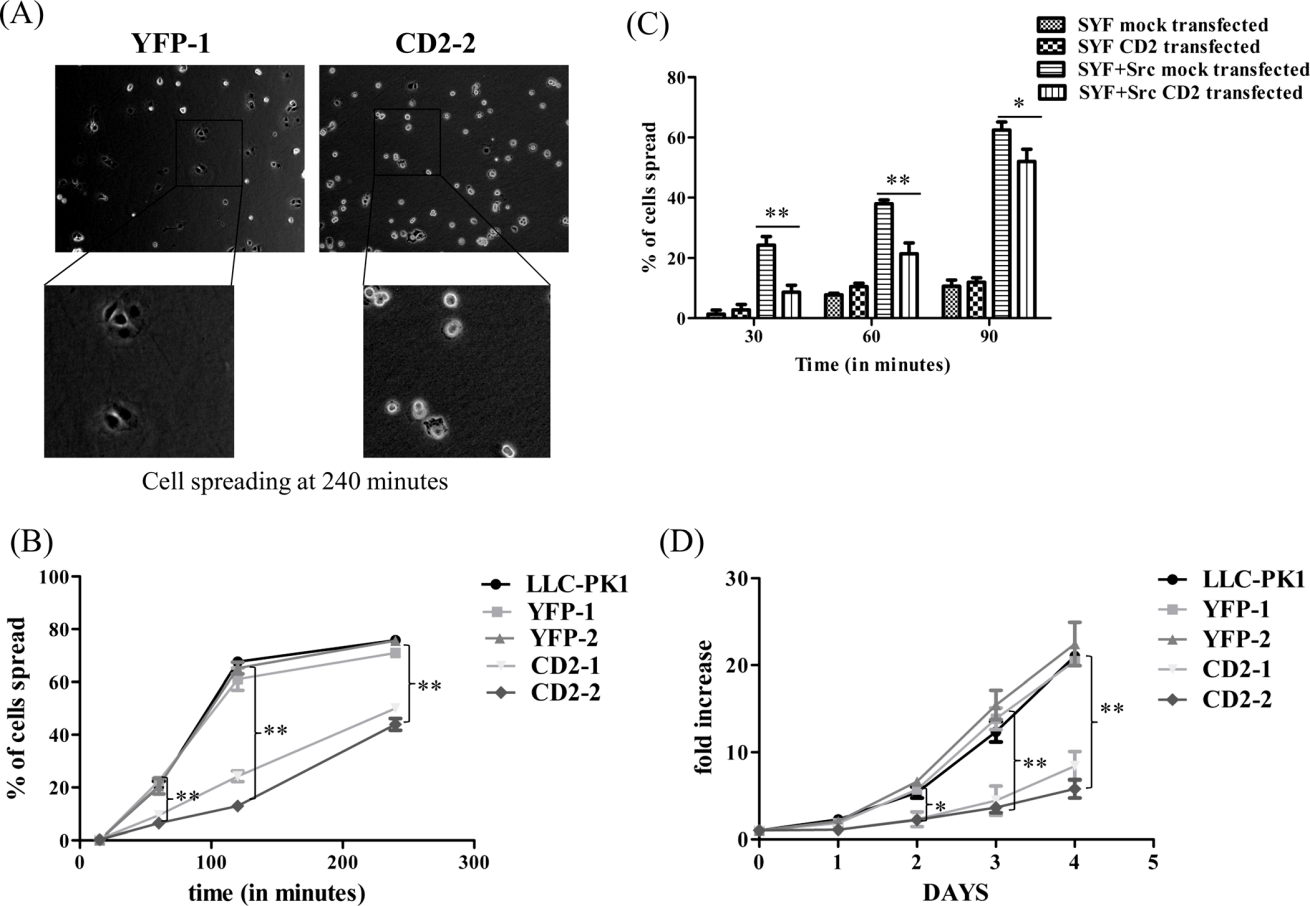
Effects of CD2 on cell spreading and proliferation (A) Top panel shows images of cell spreading after plating on dishes for 4 hours at 37°C. Larger image of cell spreading of each cell line is shown from inset panel. (B) Quantitative data show the percentage of cell spread at indicated time points. The experiment was done as described in “Materials and Methods”. Values are mean± SEM and from three independent experiments. ** p<0.01 compared with YFP-1. (C) SYF and SYF+Src cell lines were transiently transfected with YFP (mock transfected) or YFP-CD2. After visually confirming YFP expression, cells were harvested for cell spreading assay. Values are mean± SEM of three independent experiments. ** p<0.01 and *p<0.05. (D) Cell growth curve. Different cell lines were seeded at a density of 20,000 cells/well in 12 well plates, collected at indicated time points and counted as described under “Materials and Methods”. The values are mean± SEM of at least three independent experiments. *p<0.05 compared with YFP-1, **p<0.01 compared with YFP-1.

It is known that cell attachment and spreading require controlled activation and inactivation of Src kinase [3, 8, 34–36]. Thus, CD2 expression would most likely affect cell attachment and spreading if it could function as a Src SH2 domain inhibitor. Indeed, as shown in Fig 7A and 7B, cell spreading was significantly reduced in CD2-2 cells. Moreover, this defect was also observed in CD2-1 cells (Fig 7B).

In order to further test whether this is Src-specific, we measured cell spreading activity of SYF and SYF+Src cell lines that were transiently transfected with either YFP or YFP-CD2. SYF cells are mouse fibroblasts isolated from Src, Yes, and Fyn knock-out mouse embryo and SYF+Src are Src-rescued SYF cells. As depicted in Fig 7C, transient transfection of YFP-CD2 inhibited cell spreading in SYF+Src, but not in SYF cells. As expected, control experiments showed a much slower spreading of SYF cells than that of SYF+Src cells (S2 Fig).

To further reveal the importance of CD2/Src SH2 interaction in the regulation of cellular activity, we next examined whether CD2 expression inhibited cell proliferation [37, 38]. As depicted in Fig 7D, the expression of YFP-CD2 significantly reduced cell proliferation in both CD2 expressing cell lines, as compared with control.

## Discussion

Our previous *in vitro* pull-down assays indicate that the CD2 of α1 subunit of Na/K-ATPase interacts with the Src SH2 domain [14, 16]. Our new findings reveal the following. First, CD2 is indeed capable of binding to Src in live cells, and appears to be a functional ligand of Src SH2. Second, CD2 acts as a dominant negative mutant, capable of inhibiting cellular pathways and activities where Src plays an important signaling role. Finally, the interaction between the CD2 of Na/K-ATPase and Src SH2 domain appears to be essential for cardiotonic steroids to activate protein kinase cascades through the receptor Na/K-ATPase/Src complex. These and other important issues are further discussed.

### CD2 as a Src SH2 ligand

It is known that Src-mediated signal transduction is dependent not only on its kinase activity, but also on its recruitment to specific membrane assemblies through the SH2 domain [39–42]. For example, mutagenesis analyses indicate that the kinase domain and the SH2 domain cooperate in the membrane targeting of Src [6]. Blocking either kinase activity or SH2-mediated targeting to the membrane assemblies could inhibit Src-mediated signal transduction, resulting in alterations in cell spreading and growth [43–46]. Because Src plays an important role in the regulation of cell growth and migration, efforts have been made by many laboratories to develop novel Src pathway inhibitors including the identification of Src SH2 domain ligands [10, 47, 48]. Interestingly, both peptide and non-peptide ligands have been demonstrated by *in vitro* and *in vivo* studies to be effective in altering Src-mediated signal transduction [11]. For instance, there is evidence that Src SH2 domain ligands could reduce bone resorption and maybe potentially useful in the treatment of osteoporosis [49, 50]. Moreover, super-binders of SH2 domains, when expressed, could also function as antagonists of cell signaling [38].

Based on the new findings reported here, we suggest that CD2 works as a novel putative Src SH2 domain ligand [51–53]. Strikingly, its effects on Src and Src-mediated signal transduction are very much comparable to those of R175L mutant Src [43]. Although it increased basal Src activity (Fig 3), CD2 actually impeded Src-mediated signal transduction as illustrated by the inhibition of Src/FAK activation induced by plating cells onto fibronectin-coated dishes (Fig 5). This is consistent with what has been reported about the mechanism of Src/FAK interaction and their role in integrin signaling [3, 54, 55]. While CD2 expression increased phosphorylation of Src and other Src effectors like ERK and FAK, it most likely inhibited the recruitment of Src to FAK, resulting in a decrease in cell attachment-induced Src activation, and the full-activation of FAK (Fig 5) [35]. Interestingly, cell attachment-induced ERK activation was not affected by the CD2 as in the case of Src and FAK. This appears to be consistent with the reports that Src/FAK activation was not required for cell adhesion-induced ERK activation [56, 57], and provides further support that CD2 appears to be a specific Src SH2 ligand. In accordance with CD2-induced inhibition of Src/FAK signaling, it effectively reduced cell spreading and proliferation (Fig 7). In short, the new data, taken together with our previous findings [14, 16–19, 58], suggest the following unique properties of CD2 as a Src signaling pathway inhibitor. First, unlike ATP analog such as PP2, CD2 does not inhibit Src kinase activity [59]. Second, although CD2 and pNaKtide are both derived from the α1 subunit of Na/K-ATPase [18], they work differently in blocking Src-mediated signal transduction. Finally, while pNaKtide is specific for Na/K-ATPase-mediated signal transduction, CD2 appears to act as a general Src pathway inhibitor.

### Na/K-ATPase/Src interaction and its role in Src regulation

We have proposed that the α1 Na/K-ATPase interacts with Src to form a functional receptor complex for cardiotonic steroids to regulate protein kinase cascades [14, 16]. In support of our model of direct interaction between Na/K-ATPase and Src kinase, we have shown that a GST-fused CD2 interacts with Src SH2 domain whereas the N domain binds Src kinase domain [14]. Based on the latter, we have successfully delineated a 20 amino acid peptide (pNaKtide) from α1 subunit of Na/K-ATPase that is able to bind and inhibit Src kinase activity both *in vitro* and *in vivo* [18, 19]. Furthermore, when key residues in pNaKtide sequence were mutated, it gave rise to α1 mutants that pump normally but are defective in Src regulation [58]. Here, we revealed an important role of CD2-SH2 interaction in the formation of functional receptor Na/K-ATPase/Src complex. First, we found that the ectopically expressed CD2 interacted with Src as demonstrated by co-immunoprecipitation analysis (Fig 3). Second, although the expression of CD2 increased the number of Na/K-ATPase in the cell surface as revealed by ouabain binding and pumping activity assay (Fig 2), it inhibited ouabain-induced activation of Src, ERK and Akt (Fig 4). Therefore, it is concluded that CD2 domain of the α1 subunit of Na/K-ATPase is likely involved in the direct interaction with Src kinase in live cells. We further speculate that this interaction is important for the formation of a functional Na/K-ATPase/Src receptor complex through the following two possible mechanisms: (a) the binding of Src SH2 domain to the CD2 might keep Src in an active conformation, and facilitate the association of the Src kinase domain to the α1 N domain, thus helping the formation of the receptor Na/K-ATPase/Src complex; (b) because our *in vitro* binding assays (5) indicate a stronger interaction between the SH2 and CD2 than that of kinase/N domain, the CD2-SH2 association might remain even after the release of Src kinase domain from the Na/K-ATPase, resulting in a localized activation of Src and subsequent recruitment and assembly of signaling cascades in the vicinity of receptor Na/K-ATPase. Needless to say, these suggestions need to be experimentally tested.

### Uncertainties and Implications

We have inferred that the CD2 of Na/K-ATPase α1 subunit can act as a SH2 domain ligand and thereby create a deviation in phenotype of cells expressing this peptide. It is however difficult to determine whether any other Src family kinase or membrane/soluble SH2 domain protein is affected due to the expression of CD2 peptide. On the other hand, our cell spreading analyses in SYF, and SYF + Src cells provided some evidence of CD2 specificity towards Src SH2 domain. This is also supported by the fact that CD2 inhibited ouabain- and cell attachment-induced signal transduction. Furthermore, the second cytosolic domain of α1 Na/K-ATPase is known to contain a binding site for ankyrin, a cytoskeletal protein [60]. The ankyrin binding motif (MAB) expression has been shown to cause defect in membrane delivery of Na/K-ATPase in polarized epithelial cells [61]. However, it is unlikely that the ankyrin interaction is involved in the changes we reported here because of the following observations. First, the YFP-CD2 was constructed to not include the N-terminal 6 amino acid residues that are vital part of the known ankyrin binding motif [60, 61]. Second, we failed to detect any decrease in surface Na/K-ATPase as measured by ^3^H-ouabain binding. To this end, it is also of interest to mention a modest but significant increase in surface expression of Na/K-ATPase in CD2 expressing cells (Fig 2). Although it remains to be experimentally determined, it is likely that inhibition of Na/K-ATPase-mediated signal transduction by CD2 could be a reason for the observed increase [62, 63]. Finally, it remains to be determined whether CD2 binds to the bipartite phosphotyrosine binding motif on the SH2 domain [64, 65].

## Supporting Information

S1 Fig YFP-1 and CD2-1 cells were treated with ouabain in a similar manner as described in Fig 4. Cell lysates were analyzed for phospho-ERK1/2 and ERK1/2. Representative Western blot of at least three independent experiments is shown. Quantitative data were from three independent experiments and values are mean ± SEM. * p<0.05 compared with 0nM control, # p<0.05 compared with different cell line.
(TIFF)

S2 Fig Cell spreading in SYF and SYF+Src cells. **p<0.01 compared with SYF at same time point.
(TIFF)
