# Supplementary figures and images for: SH2 Ligand-Like Effects of Second Cytosolic Domain of Na/K-ATPase α1 Subunit on Src Kinase
Source: PLoS One. 2015 Nov 9;10(11):e0142119. doi: 10.1371/journal.pone.0142119 (PMC4638348; doi:10.1371/journal.pone.0142119)

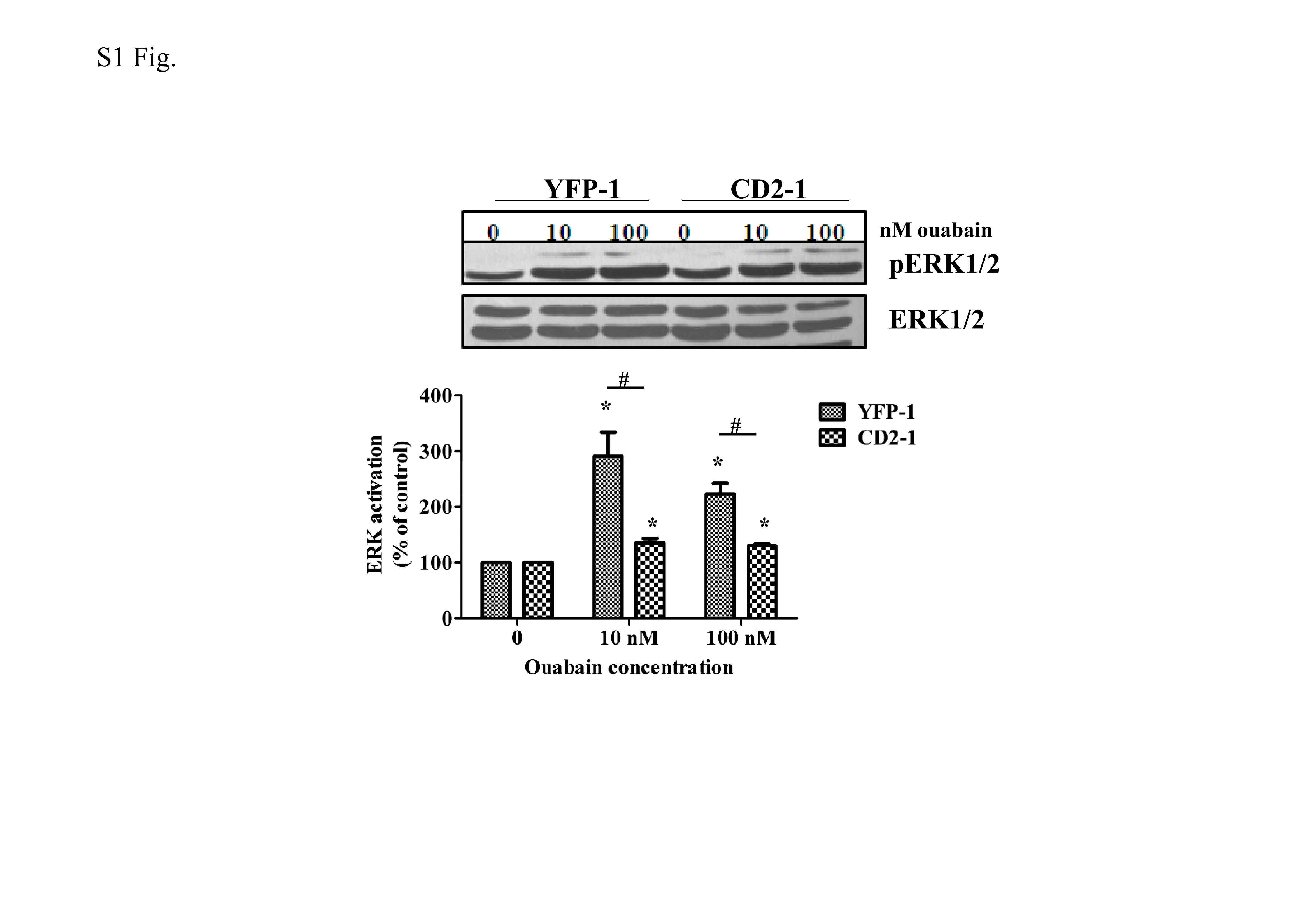

Supplement: S1 Fig — Cell lysates were analyzed for phospho-ERK1/2 and ERK1/2. Representative Western blot of at least three independent experiments is shown. Quantitative data were from three independent experiments and values are mean ± SEM. * p<0.05 compared with 0nM control, # p<0.05 compared with different cell line. (TIFF) [file pone.0142119.s001.tiff]

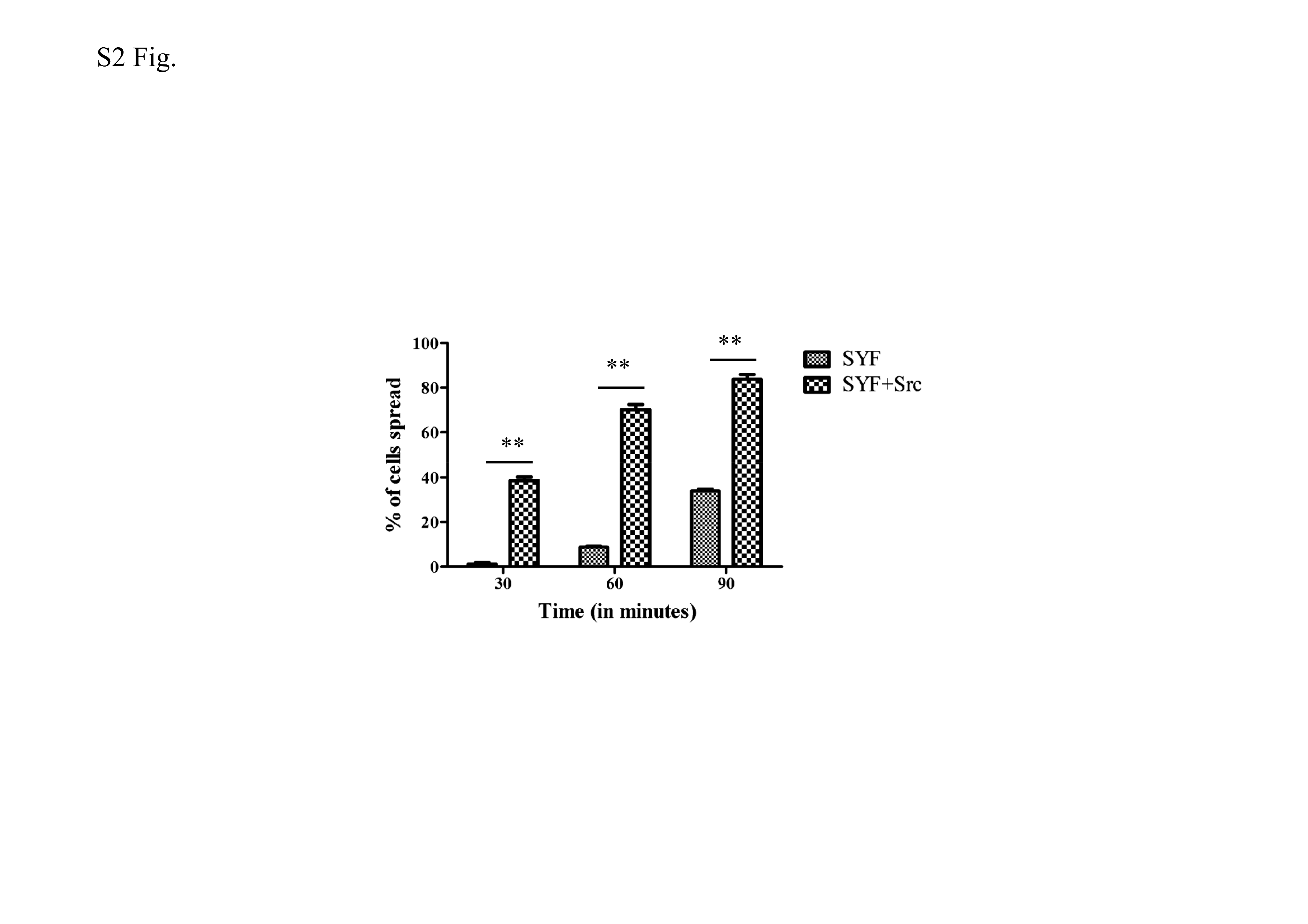

Supplement: S2 Fig — **p<0.01 compared with SYF at same time point. (TIFF) [file pone.0142119.s002.tiff]
